# Rampant C→U Hypermutation in the Genomes of SARS-CoV-2 and Other Coronaviruses: Causes and Consequences for Their Short- and Long-Term Evolutionary Trajectories

**DOI:** 10.1128/mSphere.00408-20

**Published:** 2020-06-24

**Authors:** P. Simmonds

**Affiliations:** a Nuffield Department of Medicine, University of Oxford, Oxford, United Kingdom; University Medical Center Freiburg

**Keywords:** APOBEC, COVID-19, SARS, SARS coronavirus 2, coronavirus, hypermutation, SARS-CoV-2

## Abstract

The pandemic of severe acute respiratory syndrome coronavirus 2 (SARS-CoV-2) has motivated an intensive analysis of its molecular epidemiology following its worldwide spread. To understand the early evolutionary events following its emergence, a data set of 985 complete SARS-CoV-2 sequences was assembled. Variants showed a mean of 5.5 to 9.5 nucleotide differences from each other, consistent with a midrange coronavirus substitution rate of 3 × 10^−4^ substitutions/site/year. Almost one-half of sequence changes were C→U transitions, with an 8-fold base frequency normalized directional asymmetry between C→U and U→C substitutions. Elevated ratios were observed in other recently emerged coronaviruses (SARS-CoV, Middle East respiratory syndrome [MERS]-CoV), and decreasing ratios were observed in other human coronaviruses (HCoV-NL63, -OC43, -229E, and -HKU1) proportionate to their increasing divergence. C→U transitions underpinned almost one-half of the amino acid differences between SARS-CoV-2 variants and occurred preferentially in both 5′ U/A and 3′ U/A flanking sequence contexts comparable to favored motifs of human APOBEC3 proteins. Marked base asymmetries observed in nonpandemic human coronaviruses (U ≫ A > G ≫ C) and low G+C contents may represent long-term effects of prolonged C→U hypermutation in their hosts. The evidence that much of sequence change in SARS-CoV-2 and other coronaviruses may be driven by a host APOBEC-like editing process has profound implications for understanding their short- and long-term evolution. Repeated cycles of mutation and reversion in favored mutational hot spots and the widespread occurrence of amino acid changes with no adaptive value for the virus represent a quite different paradigm of virus sequence change from neutral and Darwinian evolutionary frameworks and are not incorporated by standard models used in molecular epidemiology investigations.

**IMPORTANCE** The wealth of accurately curated sequence data for severe acute respiratory syndrome coronavirus 2 (SARS-CoV-2), its long genome, and its low substitution rate provides a relatively blank canvas with which to investigate effects of mutational and editing processes imposed by the host cell. The finding that a large proportion of sequence change in SARS-CoV-2 in the initial months of the pandemic comprised C→U mutations in a host APOBEC-like context provides evidence for a potent host-driven antiviral editing mechanism against coronaviruses more often associated with antiretroviral defense. In evolutionary terms, the contribution of biased, convergent, and context-dependent mutations to sequence change in SARS-CoV-2 is substantial, and these processes are not incorporated by standard models used in molecular epidemiology investigations.

## Abstract (precis)

The wealth of accurately curated sequence data for severe acute respiratory syndrome coronavirus 2 (SARS-CoV-2), its long genome, and its low substitution rate provides a relatively blank canvas with which to investigate effects of mutational and editing processes imposed by the host cell. The finding that a large proportion of sequence change in SARS-CoV-2 in the initial months of the pandemic comprised C→U mutations in a host APOBEC-like context provides evidence for a potent host-driven antiviral editing mechanism against coronaviruses more often associated with antiretroviral defense. In evolutionary terms, the contribution of biased, convergent, and context-dependent mutations to sequence change in SARS-CoV-2 is substantial, and these processes are not incorporated by standard models used in molecular epidemiology investigations.

## INTRODUCTION

Severe acute respiratory syndrome coronavirus 2 (SARS-CoV-2) emerged late in 2019 in the Hubei province, China, as a cause of respiratory disease occasionally leading to acute respiratory distress syndrome and death (COVID-19) (1–4). Since the first reports in December 2019, infections with SARS-CoV-2 were reported from a rapidly increasing number of countries worldwide and led to its declaration as a pandemic by the World Health Organization in March 2020. To understand the origins and transmission dynamics of SARS-CoV-2, sequencing of SARS-CoV-2 directly from samples of infected individuals worldwide has been performed on an unprecedented scale. These efforts have generated many thousands of high-quality consensus sequences spanning the length of the genome and have defined a series of geographically defined clusters that recapitulate the early routes of international spread. However, as commented elsewhere (5), there is remarkably little virus diversity at this early stage of the pandemic, and analyses of its evolutionary dynamics remain at an early stage.

The relative infrequency of substitutions is the consequence of a much lower error rate on genome copying by the viral RNA polymerase of the larger nidovirales, including coronaviruses. This is achieved through the development of a proofreading capability through mismatch detection and excision by a viral encoded exonuclease, Nsp14-ExoN (6–8). Consequently, coronaviruses show a low substitution rate over time, typically in the range of 1.5 × 10^−4^ to 10 × 10^−4^ substitutions per site per year (SSY) (9–14). Applying a midrange estimate to the 3- to 5-month timescale of the SARS-CoV-2 pandemic indicates that epidemiologically unrelated strains might show around 6 to 10 nucleotide differences from each other over the 30,000-base length of their genomes.

In the present study, we have analyzed the nature of the sequence diversity generated within the SARS-CoV-2 virus populations revealed by current and ongoing virus sequencing studies. We obtained evidence for a preponderance of driven mutational events within the short evolutionary period following the zoonotic transmission of SARS-CoV-2 into humans. Sequence substitutions were characterized by a preponderance of cytidine-to-uridine (C→U) transitions. The possibility that the initial diversity within a viral population was largely host induced would have major implications for evolutionary reconstruction of SARS-CoV-2 variants in the current pandemic as well as in our understanding both of host antiviral pathways against coronaviruses and of the longer-term shaping effects on their genome composition.

## RESULTS

### Sequence changes in SARS-CoV-2.

**FIG 1:**
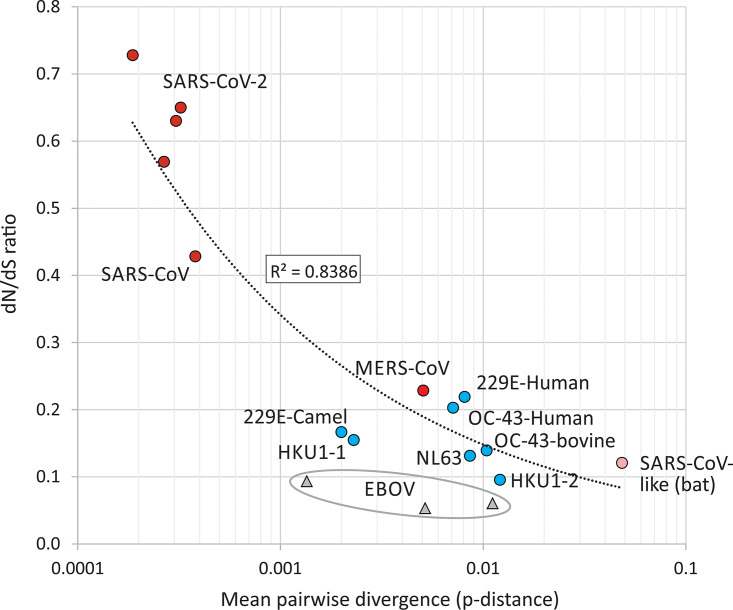
Association between sequence divergence and *dN/dS* ratio. A comparison of *dN/dS* ratios in recently emerged coronaviruses (red circles), other human coronaviruses and relatives infecting other species (blue circles), and a collection of bat sarbecoviruses (SARS-like) (pink circle). A power law line of best fit showed a significant correlation between divergence and *dN/dS* ratio (*P* = 0.000006). Sequences of the three data sets of EBOV control sequences were included (gray triangles).

Four separate data sets of full-length (near-) complete genome sequences of SARS-CoV-2 collected from the start of the pandemic to those most recently deposited on 24 April 2020 were aligned and analyzed (accession numbers listed in Table S1A in the supplemental material). Each data set showed minimal levels of sequence divergence, with mean pairwise distances ranging from 5.5 to 9.5 nucleotide differences between each sequence. However, several aspects of the frequencies and sequence contexts of the observed changes were unexpected. First, the ratio of nonsynonymous (amino acid changing) to synonymous substitutions (*dN*/*dS*) was high, in the range of 0.57 to 0.73 among the different SARS-CoV-2 data sets. This contrasts with a much lower ratio (consistently below <0.22) in sequence data sets assembled for the other human coronaviruses (Table 1). Including a range of coronaviruses in the analysis, there was a consistent association between *dN*/*dS* ratios and the degree of sequence divergence (Fig. 1).

**TABLE 1:** Coronavirus sequence data sets used for the study

Virus	No.	Length (bp)	MPD^*a*^	*dN/dS*^*b*^
Zoonotic coronaviruses				
SARS-CoV-2_Charite	115	29748	0.000187	0.728
SARS-CoV-2_Repl1	300	29409	0.000267	0.569
SARS-CoV-2_Repl2	300	29408	0.000306	0.630
SARS-CoV-2_Repl3	286	29404	0.000322	0.650
SARS-CoV-1-like (bat)	40	29480	0.048414	0.121
SARS-CoV-1	22	29443	0.000381	0.428
MERS-CoV	26	30043	0.005065	0.228
Other human and related coronaviruses				
OC43-human	178	30135	0.0081	0.219
OC43-bovine	113	30485	0.0104	0.139
HKU1-gt1	27	29613	0.0023	0.155
HKU1-gt2	12	29610	0.0121	0.096
NL63	61	27453	0.0086	0.131
229E-human	26	26846	0.0071	0.203
229E-camel	33	27051	0.002	0.167

a Mean nucleotide *p* distances between complete genome sequences.

b Frequency of nonsynonymous (*dN*) to synonymous (*dS*) *p* distances.

10.1128/mSphere.00408-20.2 TABLE S1 Listing of SARS-CoV-2 and EBOV sequences analyzed in the study. Download Table S1, DOCX file, 0.1 MB. Copyright © 2020 Simmonds. 2020 Simmonds This content is distributed under the terms of the Creative Commons Attribution 4.0 International license.

We next estimated the frequencies of individual transitions and transversions occurring during the short-term evolution of SARS-CoV-2. Sequence differences between each SARS-CoV-2 full-genome sequence and a majority rule consensus sequence generated for each of the four SARS-CoV-2 data sets were calculated. The directionality of sequence change underlying the observed substitutions was inferred by restricting the analysis to polymorphic sites with a minimal number of variable bases (typically singletons). In practice, because of the scarcity of substitutions, variability thresholds of 10%, 5%, 2%, and 1% yielded similar numbers and relative frequencies of each transition and transversion. Equivalent evidence for directionality was obtained through comparison of each sequence in the data set with the first outbreak sequence (MN908947; Wuhan-Hu-1), approximately ancestral to the currently circulating SARS-CoV-2 strains (data not shown). For the purposes of the analysis presented here, a consensus-based 5% threshold was used.

**FIG 2:**
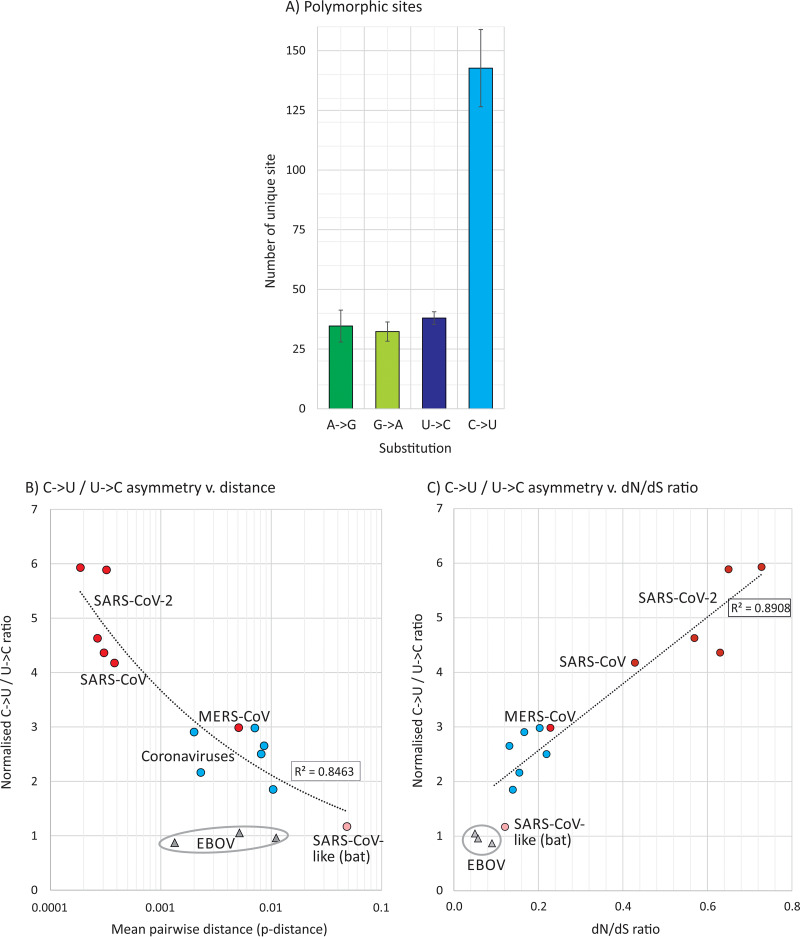
Association of excess C→U transitions with divergence. (A) Numbers of sites in the SARS-CoV-2 genome with each of the four transitions. Bar heights represent the means from the three sequence samples; error bars show one standard deviation (SD). (B) Relationship between sequence diversity and a normalized metric of asymmetry between the numbers of C→U and U→C transitions (where 1.0 is the expected number). Power law regression line was significant at a *P* value of <0.0001. (C) Association of *dN/dS* ratio with C→U/U→C asymmetry. The power law regression lines were significant at *P* values of 0.001 and 0.0004, respectively. Points are colored as in Fig. 1.

A listing of the sequence changes revealed a striking (approximately 4-fold) excess of sites where C→U substitutions occurred in SARS-CoV-2 sequences compared to the other three transitions (Fig. 2A). This excess was the more remarkable given there was an almost 2-fold greater number of U bases in the SARS-CoV-2 genome than Cs (32.1% compared to 18.4%, respectively). To formally analyze the excess of C→U transitions, we calculated an index of asymmetry (frequency [f][C→U]/f[U→C]) × (fU/fC) and compared this with degrees of sequence divergence and *dN*/*dS* ratios in SARS-CoV-2 and other coronavirus data sets (Fig. 2B and C). This comparison showed that the excess of C→U substitutions was most marked among very recently diverged sequences associated with the SARS-CoV-2 and SARS-CoV outbreaks and was reduced significantly in sequence data sets of the more divergent human coronaviruses (NL63, OC43, 229E, and OC43) as sequences accumulated substitutions.

A parallel analysis of the full-genome sequences of Ebola virus (EBOV) was performed to determine whether the compositional abnormalities observed in SARS-CoV-2 arose as artifacts of the next-generation sequencing (NGS) methods used to generate the data or indeed occurred in a different RNA virus with distinct entry, replication, and packaging strategies. Available sequences of EBOV on GenBank were divided into three groups, corresponding to those associated with the most recent outbreak in the Congo, in Sierra Leone, and elsewhere in West Africa in the 2014 outbreak, and finally a collection of older strains (see Table S1B). These showed mean levels of within-group sequence divergence of 0.1%, 1.1%, and 0.5%, respectively, spanning the range of divergences in the analyzed SARS-CoV-2 and other coronavirus data sets. In marked contrast to that of SARS-CoV-2, sequences consistently showed *dN/dS* ratios of <0.1 (Fig. 1) and no mutational asymmetry of C→U/U→C (Fig. 2B), irrespective of their sequence divergence.

**FIG 3:**
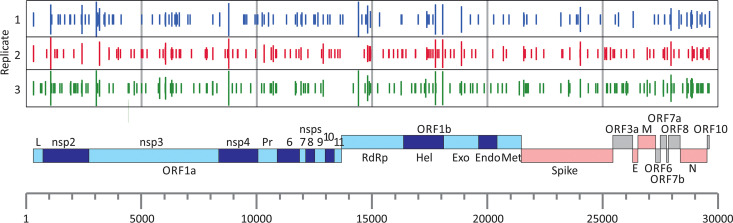
Positions of C→U transitions in the SARS-CoV-2 genome in each of the three replicate SARS-CoV-2 sequence data sets were matched to a genome diagram of SARS-CoV-2 (using the annotation from the prototype sequence MN908947). The numbers of transitions at each site are shown on a log scale, with the shortest bars indicating individual substitutions.

**FIG 4:**
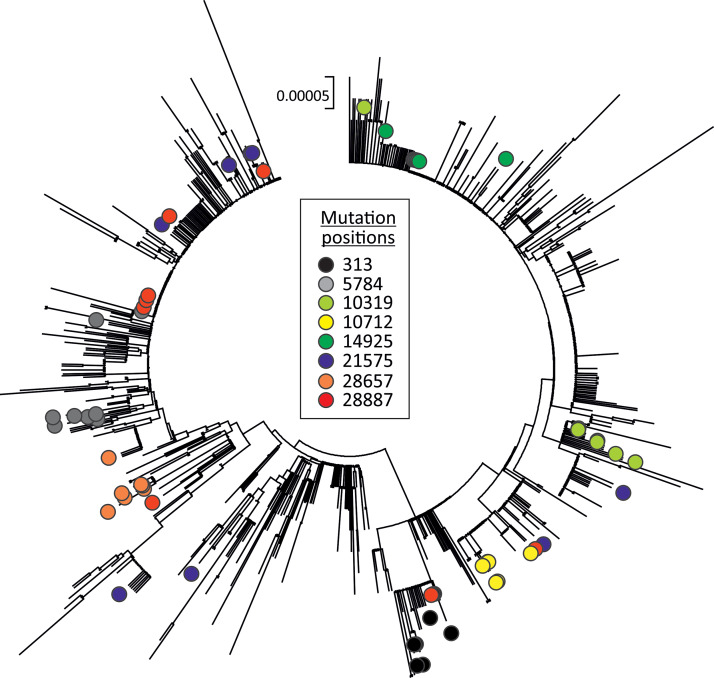
Phylogeny of SARS-CoV-2 and positions of sequences with C→U changes. A neighbor-joining tree of 865 SARS-CoV-2 complete genome sequences was constructed in MEGA6 (41). Labels show the position of sequences containing a selection of C→U transitions at the genome positions indicated in the key.

C→U substitutions were scattered throughout the SARS-CoV-2 genome (Fig. 3). Long bars representing more polymorphic sites were frequently shared between replicate data sets, but unique substitutions (occurring once in the data set [short bars]) showed largely separate distributions. Substitutions were not focused toward any particular gene or intergenic region, although all three data sets showed marginally higher frequencies of substitutions in the N gene. A selection of sequences showing C→U changes in different genome regions was plotted in a phylogenetic tree containing sequences from the SARS-CoV-2 data set (Fig. 4). With the resolution possible in the tree generated from such a minimally divergent data set, many sequences with shared C→U changes were not monophyletic (e.g., those with substitutions at positions 5784, 10319, 21575, 28657, and 28887). This lack of grouping is consistent with multiple *de novo* occurrences of the same mutation in different SARS-CoV-2 lineages.

**FIG 5:**
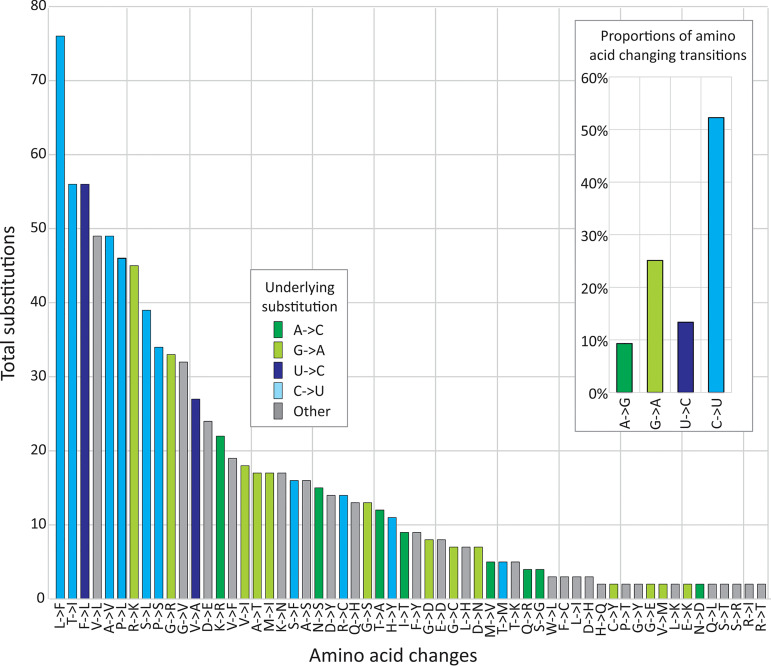
Amino acid changes induced by different nucleotide substitutions. Numbers of individual amino acids changes observed in the combined SARS-CoV-2 data set (864 sequences) at a 5% variability threshold. Bars are colored based on the underlying nucleotide changes. Inset graph shows the relative proportions of transitions leading to amino acid changes.

The abnormally high *dN/dS* ratios of 0.6 to 0.7 in SARS-CoV-2 sequences (Table 1; Fig. 1) indicated that around 50% of nucleotide substitutions would produce amino acid changes (if approximately 75% of nucleotide changes are nonsynonymous). On analysis of amino acid sequence changes, a remarkable 52% of nonsynonymous transitions in the SARS-CoV-2 sequence data set were the consequence of C→U transitions (Fig. 5), compared to 26%, 10%, and 7% for G→A, U→C, and A→C transitions, respectively. These ratios are comparable to those at all sites (Fig. 1) apart from the greater proportion of nonsynonymous G→A changes. Some variability might be expected given the potential fitness effects of specific amino acid changes and their likelihood of fixation. Notwithstanding this, the underlying mechanism that leads to C→U hypermutation therefore also drives much of the amino acid sequence diversity observed in SARS-CoV-2.

**FIG 6:**
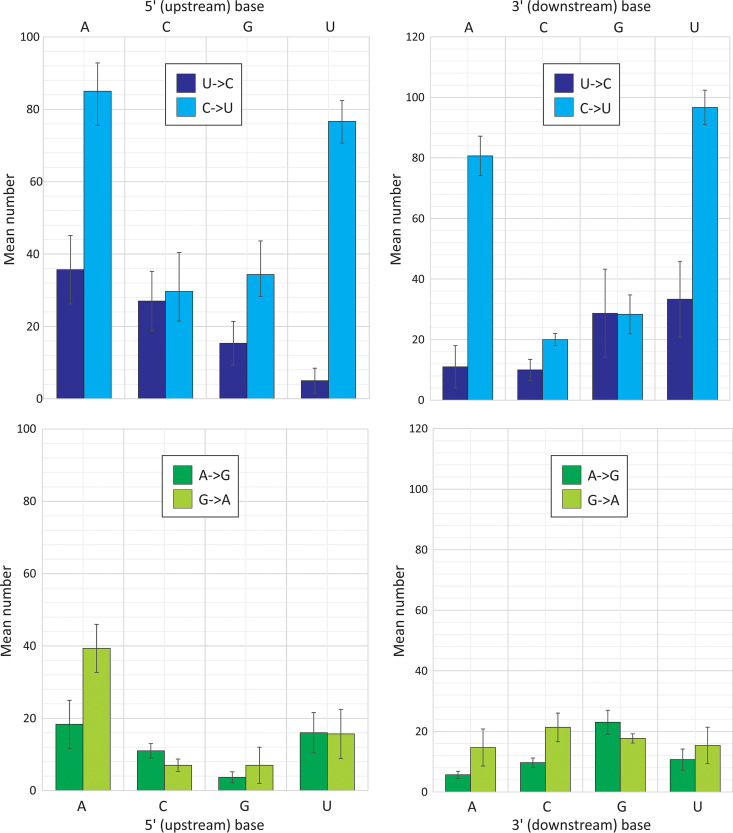
Influence of 5′ and 3′ base contexts on C↔U and G↔A transition frequencies. Totals of each transition in the SARS-CoV-2 sequence data set split into subtotals based on the identity of the 5′ (left) and 3′ (right) base. Bar heights represent the means from the three sequence samples; error bars show standard deviations. A further division into the 16 combinations of 5′ and 3′ base contexts is provided in Fig. S1 in the supplemental material.

The context of cytidines within a sequence strongly influenced the likelihood of it mutating to a U (Fig. 6). The greatest numbers of mutations were observed if the upstream (5′) base was an A or U. There was also a similar approximately 4-fold increase in transitions if these bases were located on the downstream (3′) side. The effects of the 5′ and 3′ contexts were additive: C residues surrounded by an A or U at both 5′ and 3′ sides were 10-fold more likely to mutate than those flanked by C or G residues (mean of 31.9 transitions compared to 3.6). Splitting the data down into the 16 combinations of 5′ and 3′ contexts, a 5′ U far more potently restricted non-C→U substitutions than a 5′ A (see Fig. S1), while 5′ G or 5′ C almost eliminated substitutions irrespective of the 3′ context. No context created any substantial asymmetry in G→A compared to A→G transitions.

10.1128/mSphere.00408-20.1 FIG S1 Frequencies of transitions in all 5′ and 3′ base context combinations. Effects of 5′ and 3′ bases on transition frequencies in SARS-CoV-2 full-genome sequences. Download FIG S1, DOCX file, 0.3 MB. Copyright © 2020 Simmonds. 2020 Simmonds This content is distributed under the terms of the Creative Commons Attribution 4.0 International license.

**FIG 7:**
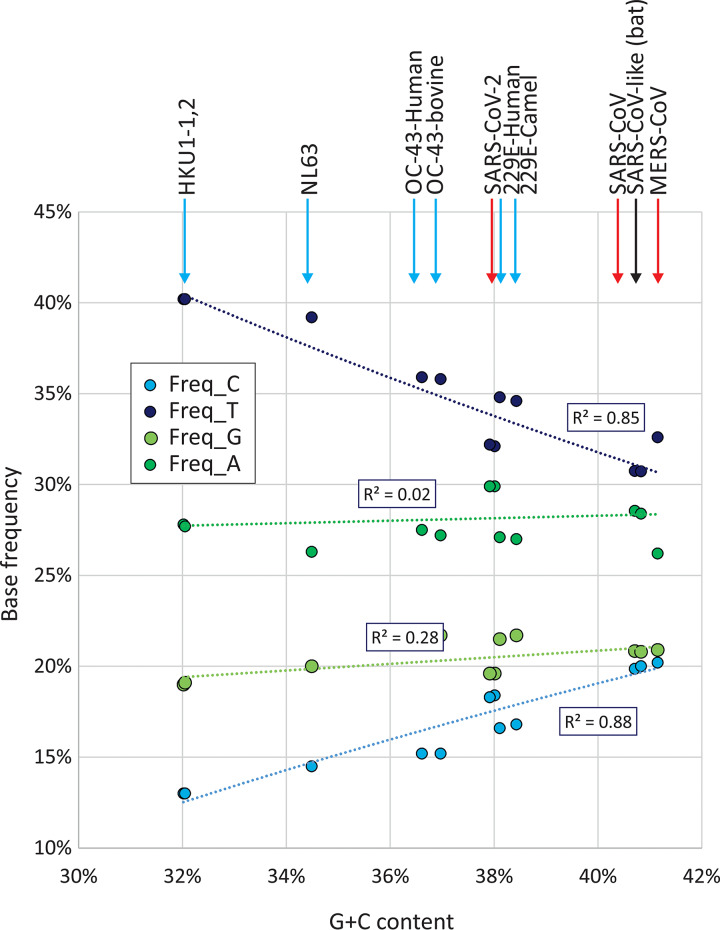
Base frequencies in different coronaviruses. Relationship between G+C content and frequencies of individual bases in coronaviruses. The associations between C depletion and U enrichment with G+C content were both significant by linear regression at *P* = 5 × 10^−7^ and *P* = 5 × 10^−6^, respectively. No significant associations were observed between G+C content and G (*P* = 0.05) or A (*P* = 0.62) frequencies. Arrows are color coded as for Fig. 1.

**FIG 8:**
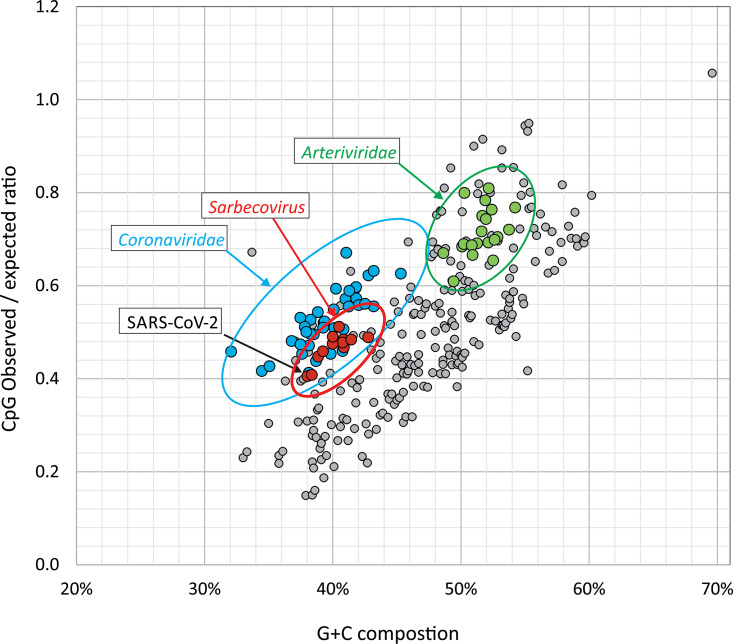
Suppression of CpG dinucleotides in SARS-CoV-2 and other coronaviruses. Comparison of CpG frequencies of SARS-CoV-2, other coronaviruses, and a set of other mammalian RNA viruses; each data point represents an individual currently classified species; accession numbers are listed in Table S1. CpG frequencies were expressed as the ratio of their observed frequency to the expected frequency based on their G+C content (*y* axis).

The G+C content of coronaviruses varied substantially between species, with highest frequencies in the recently emerged zoonotic coronaviruses (Middle East respiratory syndrome [MERS]-CoV, 41%; SARS-CoV, 41%; and SARS-CoV-2, 38%) and lowest in HKU1 (32%). Collectively, there was a significant relationship between C depletion and U enrichment with G+C content (Fig. 7). The difference in G+C content was indeed almost entirely attributable to changes in the frequencies of C and U bases: the 9% difference in G+C content between MERS-CoV and HKU1 arose primarily from the 20% to 13% reduction in frequencies of C. There was a comparable 8% increase in the frequency of U. Their combined effects left frequencies of G and A relatively unchanged. It has been proposed that the asymmetry in C and U frequencies may originate in part through the selective loss of CpG dinucleotides in the genome (15). To investigate this, the degree of suppression in SARS-CoV-2, other sarbecoviruses, and other coronaviruses was compared with representative sequences of each currently classified mammalian RNA virus species (excluding double-stranded RNA [dsRNA] viruses). Mammalian RNA viruses (Fig. 8, gray circles) demonstrate the previously described relationship between G+C content and CpG suppression (16). The data points for the separately labeled SARS-CoV-2, other SARS-like viruses in bats (sarbecoviruses; red), and the remainder of the coronaviruses (blue) and arteriviruses (green) overlap these values (Fig. 8). Overall, SARS-CoV-2 and other coronaviruses actually show less suppression of CpG for a given G+C content than is typical for other RNA viruses. SARS-CoV-2 and other coronaviruses are therefore not compositionally unusual by these metrics, providing no evidence that CpG suppression *per se* is associated with their mutational and compositional abnormalities.

## DISCUSSION

The wealth of sequence data generated since the outset of the SARS-CoV-2 pandemic, the accuracy of the sequences obtained by a range of NGS technologies, and the long genomes and very low substitution rate of coronaviruses provided a unique opportunity to investigate sequence diversification at very high resolution. The findings additionally provide insights into the mutational mechanisms and contexts where sequence changes occur. Third, it informs us about the longer-term evolution of viruses and potential effects of the cell in molding virus composition.

### The mechanism of SARS-CoV-2 hypermutation.

The most striking finding that emerged from the analysis of more than 1,000 SARS-CoV-2 genomes was the preponderance of C→U transitions compared to other substitutions in the initial 4 to 5 months of its evolution. These accounted for 38% to 42% of all changes in the four SARS-CoV-2 data sets. In seeking alternative nonbiological explanations for this observation, they cannot have arisen through misincorporation errors in the nex-generation sequence methods used to produce the data set because the analysis in the present study was restricted to consensus sequences. These are generally assembled from libraries that typically possess reasonable coverage and read depth; error frequencies of <10^−4^ per site (17) would therefore improbably create a consensus change in a sequence library. There was, furthermore, no comparable increase in G→A mutations (Fig. 2), and the sequence context in which sequencing errors occur (a 5′ or 3′ C or G [17]) did not match the favored context for mutation observed in our data set (Fig. 6). Finally, the parallel analysis of a similarly large collection of consensus sequences of EBOV demonstrated very low *dN/dS* values and no excess of C→U mutations over U→C (Fig. 1 and 2). As EBOV and SARS-CoV-2 data were generated by comparable NGS methods, it is therefore extremely unlikely that the mutational and compositional abnormalities of SARS-CoV-2 described in the study were the results of methodological artifacts.

This asymmetric mutation, furthermore, cannot have arisen through a mutational effect of the coronavirus RNA-dependent RNA polymerase during virus replication. By definition, a coronavirus RNA genome descends from any other through an equal number of copies of the positive and negative strands—any tendency to misincorporate a U instead of a C would be reflected in a parallel number of G→A mutations where this error occurred on the minus strand (or vice versa). As demonstrated, however, G→A mutations occurred at a much lower frequency than C→U mutations and similarly to A→G (Fig. 2A and 6).

The most cogent explanation for C→U hypermutation is the action of RNA editing processes within the infected cell. A well-characterized antiviral pathway involves the interferon-inducible isoform of adenosine deaminase acting on RNA type 1 (ADAR1) (18). This edits A to inosine in regions of viral double-stranded RNA, which is subsequently copied as a G. Irrespective of its widely demonstrated antiviral role in a range of typically minus-stranded RNA viruses, the mutations it creates do not match those observed in SARS-CoV-2 or other coronaviruses. First, ADAR1 targets dsRNA, and so editing effects tend to be symmetric with A→G substitutions being matched by U→C mutations. Second, the direction of mutation is wrong. The focus of the analysis in the present study was on infrequent or unique polymorphisms where ancestral and mutant bases can be inferred. The excess of C→U transitions is the opposite of those induced by ADAR1.

A second interferon-inducible pathway edits retroviral DNA during transcription and is strand specific; its typical antiviral activity is to mutate single-stranded proviral DNA formed after first-strand synthesis from genomic RNA (19–21). The deamination of Cs to Ts leads to the observed excess of G→A changes in the complementary positive-stranded RNA virus genome (22). This editing function is performed by members of the apolipoprotein B mRNA-editing enzyme, catalytic polypeptide-like (APOBEC) family, many of which possess defined antiviral functions against retroviruses, hepatitis B viruses, small DNA viruses, and intracellular mobile retroelements (reviewed in reference 23). The APOBEC3 gene family members that are primarily involved in antiviral defense show evidence of extensive positive selection and expansion over the course of mammalian evolution, particularly in the primate lineage. Humans possess 7 active antiviral proteins (A3A, A3B, A3C, A3D, A3F, A3G, and A3H) that contrast with the single A1 gene in rodents and a range of other mammals (24–26). Other mammals possess a diverse range of largely independently duplicated APOBEC3 genes, with four paralogs in cats, three in cows and sheep, six in horses, and often more than 10 in different bat species. However, their comparative activities and editing capabilities for different DNA and RNA substrates remain functionally largely unexplored.

While deamination of cytidines in single-stranded DNA sequences is a hallmark of APOBEC function, APOBECs show binding affinities for single-stranded RNA templates that may mediate antiviral functions. A3B and A3F have been shown to block retrotransposition of a LINE-1 transposon mRNA through a nondeamination pathway (27), potentially through binding to single-stranded RNA. Direct editing of HIV-1 RNA by the rat A1 APOBEC and the accumulation of C→U hypermutation verified that RNA could also be used as a substrate for deamination (28). This suggested to the authors at that time that APOBEC-mediated RNA editing was a potential antiviral activity mechanism against RNA viruses as well as retroviruses.

Since then, evidence supporting this conjecture has been difficult to obtain; the virus inhibitory effect of APOBECs against enterovirus A71, measles, mumps, and respiratory syncytial viruses were not shown to be associated with the development of virus mutations (29, 30). Similarly, A3C, A3F, or A3H, but not A3A, A3D, and A3G, were shown to inhibit the replication of the human coronavirus, HCoV-NL63, but their expression did not lead to *de novo* C→U (or G→A) mutations on virus passaging (31). On the other hand, it has been demonstrated that A3A and A3G possess potent RNA editing capability on mRNA expressed in hypoxic macrophages (32), natural killer cells (33), and transfected A3G-overexpressing HEK 293T cells (34). These latter findings verify that APOBECs possess RNA editing capabilities but do not provide any mechanistic context for the potential inhibition of RNA virus replication by this mechanism. Nevertheless, the pronounced asymmetry in C→U transitions in SARS-CoV-2 and the preferential substitution of Cs flanked by U and A bases on both 5′ and 3′ sides (Fig. 6) that broadly matches what is known about the favored contexts of A3A, A3F, and A3H (35) provides strong circumstantial grounds for suspecting a role of one or more APOBEC proteins in coronavirus mutagenesis.

The findings of C→U mutations at the consensus genome sequence level have also been observed within virus populations in a recent analysis of intrahost sequence diversity in lung-derived COVID-19 samples (36). Mutations showed the 5′ and 3′ A/U contexts as observed in the present study and were proposed by the authors as representing editing sites for APOBEC1. Intrahost diversity in this study was, however, dominated by minor populations generated from G→A and U→C substitutions; their symmetry and lack of 5′ or 3′ context led the authors to propose the editing effects of ADAR in viral dsRNA. These and other mutations such as A→U and U→A transversions mediated through an as-yet-uncharacterized mechanism hint at the complexity of host effects on virus sequence change. The combination of exceptionally long genomes (≈30,000 bases), an otherwise low mutation rate, and the unprecedented size of the present data set of accurate minimally divergent SARS-CoV-2 sequences assembled postpandemic has enabled these mutational signatures to be so clearly observed. RNA editing may indeed represent a powerful antiviral mechanism with potentially lethal effects of even single mutations introduced into the genomic sequence. These make APOBEC-mediated anticoronaviral activity plausible in virological terms.

### Evolutionary implications.

The key findings in the study were the combined evidence for an APOBEC-like editing process driving initial sequences changes in SARS-CoV-2 and that the observed substitutions have not arisen through a typical pattern on random mutation and fixation that is assumed in evolutionary models. A specific problem for evolutionary reconstructions would be the existence of highly uneven substitution rates at different sites; APOBEC-mediated editing (and indeed the pattern of C→U transition in SARS-CoV-2 sequences) is strongly dependent on sequence context and, for at least two APOBECs, additionally influenced by their proximity to RNA secondary structure elements in the target sequence (32, 35). Sequence changes in SARS-CoV-2 and other coronavirus genomes may therefore be partially or largely restricted to a number of mutational hot spots that may promote convergent changes between otherwise genetically unlinked strains. As demonstrated in Fig. 4, these can conflict with relationships reconstructed from phylogenetically informative sites. Furthermore, the substitution rate reconstructed for SARS-CoV-2 and potentially other coronaviruses may represent an uncomfortable amalgam of both the accumulation of neutral changes and forced changes induced by APOBEC-like editing processes that may obscure temporal reconstructions. A recent analysis of SARS-CoV-2 genomes illustrates these problems (5); only a tiny fraction of variable sites (0.34%) were found to phylogenetically informative, while a high frequency of unresolved quartets demonstrates further the lack of phylogenetic signal in SARS-CoV-2 evolution reconstructions. The occurrence of multiple driven changes under host-induced selection is consistent with these cautionary observations.

The other important consequence of C→U hypermutation is that most of the amino acid sequence diversity observed in SARS-CoV-2 strains originates directly from forced mutations and therefore cannot be regarded in any way as adaptive for the virus (Fig. 5). An RNA editing mechanism of the type discussed above evidently places a huge mutational load on SARS-CoV-2 that may underpin the abnormally high *dN/dS* ratios recorded in SARS-CoV-2 and SARS-CoV sequence data sets (Fig. 1). It is likely that many or most amino acid changes are mildly deleterious and transient; repeated rounds of mutation at favored editing sites followed by reversion may therefore contribute to the large numbers of scattered substitutions in SARS-CoV-2 sequences that conflict with their phylogeny.

Finally, it is intriguing to speculate on the long-term effects of the C→U/U→C asymmetry and the extent to which this may contribute to the previously described compositional abnormalities of coronaviruses (15, 37). As described above in connection with mutation frequencies, the compositional asymmetries cannot directly arise through viral RdRp mutational biases, because any resulting base frequency differences would be symmetric (i.e., G ≈ C and A ≈ U). Instead, it appears that the observed imbalances in frequencies of complementary bases reflect the progressive depletion of C residues and accumulation of Us by the APOBEC-like mutational process on the genomic (+) strand of coronaviruses. Culminating in the compositionally highly abnormal HKU1 sequences (15), this appears to have driven the G+C content of coronaviruses as low as 32% while remarkably leaving G and A frequencies more or less unaltered (Fig. 7). Intriguingly, the bat-derived coronaviruses along with the recently zoonotically transferred viruses into humans show the least degree of compositional asymmetry.

The expansions in APOBEC gene numbers, extensive positive selection, and the consequent variability in APOBEC nucleic acid targeting (23) may indeed create distinct selection pressures on coronaviruses in different hosts. The immediate appearance of C→U hypermutation in SARS-CoV-2 and SARS-CoV genomes in humans may therefore represent the initial effects of replication in a more hostile internal cellular environment than that found in what might be a better coadapted virus-tolerized immune system of a bat (38). Zoonotic origins are suspected for other human coronaviruses but at more remote times (39); perhaps they have taken their mutational and adaptive journeys already.

## MATERIALS AND METHODS

### SARS-CoV-2 and other coronavirus data sets.

The 1,000 closest matched sequences to the prototype strain of SARS-CoV-2, NC_045512, were downloaded on 24 April 2020. Sequences with large internal gaps, ambiguous bases, and other markers of poor sequence quality were excluded, leaving a total of 865 sequences for analysis. These were divided into three data samples, corresponding to sequences 1 to 300, 301 to 600, and 601 to 865 (sequences listed in Table S1A in the supplemental material). An additional data set of 117 well-curated SARS-CoV-2 sequences was downloaded from Konsiliarlabor für Coronaviren (https://civnb.info/sequences/) on 13 April 2020 and represents a further independent sample set. A listing of further data sets of SARS-CoV, MERS-CoV, and other human coronaviruses is provided in Table 1. All available complete genome sequences of EBOV were downloaded from GenBank on 3 May 2020, of which 1,193 were used for mutational analysis after removal of incomplete, poor quality, and synthetic sequences (Table S1B).

### Sequence analysis.

Calculation of pairwise distances, nucleotide composition, and listing of sequence changes were performed using the SSE package version 1.4 (http://www.virus-evolution.org/Downloads/Software/) (40).

10.1128/mSphere.00408-20.3 TABLE S2 Sequences used for comparison of suppression of CpG in coronaviruses. Download Table S2, DOCX file, 0.1 MB. Copyright © 2020 Simmonds. 2020 Simmonds This content is distributed under the terms of the Creative Commons Attribution 4.0 International license.
