# Supplementary material for: Rampant C→U Hypermutation in the Genomes of SARS-CoV-2 and Other Coronaviruses: Causes and Consequences for Their Short- and Long-Term Evolutionary Trajectories
Source: mSphere. 2020 Jun 24;5(3):e00408-20. doi: 10.1128/mSphere.00408-20 (PMC7316492; doi:10.1128/mSphere.00408-20)
Supplement: TABLE S1 [file mSphere.00408-20-st001.docx]

SUPPLEMENTARY DATA

TABLE S1

LISTING OF SARS-CoV-2 SEQUENCES ANALYSED IN THE STUDY

**Set 1** (n = 300)

MT359866, MT276329, MT152824, MT295465, MT325597, MT328035, MT358401, MT350240, MT322417, MT322419, MT322418, MT350269, MT328034, MT359865, MT293219, MT344948, MT325599, MT304488, MT276323, MT246462, MT246452, NC_045512, MT121215, MT019531, LC529905, MT334529, MT135041, MT019529, MT108784, MN996528, MT135043, MT049951, MT019532, MT114413, MT192772, MT350282, MT339041, MT324062, MT281577, MT163719, MT246480, MT114412, MT114418, MT334533, MT334542, MT322401, MT334541, MT263396, MT251978, MT322424, MT246460, MT114417, MT322405, MT358637, MT334544, MT334538, MT326187, MT163718, MT358675, MT159710, MT192773, MT114415, MT114414, MT339039, MN988668, MT358705, MT334547, MT328032, MT039890, MT019533, MT334534, MT276324, MT258381, MT184912, MT159715, MT159709, MT159708, MT159707, MT159706, MT118835, MT106053, MT304483, MT358715, MT334540, MT326178, MN975262, MT322413, MN994468, MT326190, MT295464, MT163717, MT159722, MT159718, MT159717, MT027064, MT259278, MT246466, MT263381, MT322409, MT263395, MT325634, MT325593, MT325592, MT326117, MT304487, MT276328, MT259254, MT159720, MT159712, MT159705, MT123290, MT027062, MN985325, MT358702, MT019530, MT230904, MT258380, MT007544, MT358736, MT263429, MT258377, MT350239, MT345819, MT325621, MT325620, MT325619, MT325617, MT258378, MT123291, MT163716, MT106052, MN997409, MT345877, MT246478, MT246467, MT358402, MT184913, MT039887, MT276331, LC534418, MT039888, MN988713, MT350280, MT350279, MT350274, MT344946, MT325640, MT325618, MT325615, MT325614, MT325609, MT325591, MT325576, MT325566, MT322410, MT304476, MT304475, MT304474, MT262900, MT184908, MT358694, MT358649, MT325579, MT258379, MT251976, MT246454, MT344961, MT345875, MT322422, MT293204, MT093571, MT358695, MT325613, MT325563, MT325562, MT322397, LC534419, LR757996, MT358744, MT358740, MT345840, MT345834, MT326092, MT350278, MT350277, MT350276, MT350273, MT350272, MT350270, MT350267, MT350266, MT350265, MT350264, MT350263, MT325638, MT325637, MT325633, MT325632, MT325631, MT325630, MT325627, MT325626, MT325616, MT325611, MT325605, MT325603, MT325601, MT325600, MT325598, MT325594, MT325590, MT325583, MT325582, MT325580, MT325578, MT325565, MT325561, MT304486, MT276326, MT262915, MT262899, MT262896, MT246459, MT344962, MT326052, MT324684, MT322402, MT263468, MT358742, MT126808, LR757995, MT344959, MT344949, MT344947, MT345869, MT325602, MT325585, MT325584, MT184910, MT325574, MT258383, MT066176, MT066175, MT350275, MT350268, MT344960, MT325639, MT325636, MT325635, MT325629, MT325628, MT325587, MT325586, MT325577, MT325568, MT304490, MT106054, MT044257, MN994467, MT350251, MT325612, LC528232, MT334546, MT325564, MT304478, MT184911, LC528233, MT358743, MT350271, MT344954, MT325622, MT325595, MT326167, MT304484, MT304479, MT276327, MT192759, MT358716, MT350253, MT344944, MT325610, MT325589, MT123292, MT358718, MT345882, MT259230, LR757998, MT345806, MT325569, MT263459, MT293159, MT259231, MT066156, MT114416, MT344953, MT344945, MT325624, MT325623, MT325606, MT325596

**Set 2** (n = 300)

MT325588, MT350254, MT276598, MT350255, MT246455, MT246461, MT345887, MT358669, MT326103, MT291836, MT350257, MT325581, MT263406, MT293220, MT326096, MT246484, MT350247, MT322406, MT350236, MT322415, MT350237, MT322416, MT345888, MT263458, MT263435, MT293218, MT293166, MT263404, MT293183, MT293160, MT325570, MT304491, MT304489, MT304480, MT291831, MT259269, MT325604, MT304477, MT259229, MT345868, MT345841, MT246474, MT246667, MT123293, MT114419, MT345873, MT326100, MT039874, MT291827, MT093631, MT350252, MT325625, MT325607, MT325575, MT325572, MT325571, MT322423, MT263421, MT358722, MT344963, MT334535, MT325567, MT291828, MT328033, MT259273, MT246450, MT358706, MT325608, MT325573, MT326132, MT259236, MN996530, MT263438, MT263437, MT358748, MT259226, MT350245, MT350244, MT344958, MT344956, MT344955, MT291834, MT293210, MT326065, MT322395, MN996531, MT345871, MT345803, MT358696, MT326153, MT322408, MT293202, MT293181, MT263399, MT246481, MT246477, MT358741, MT263446, MT344957, MT326129, MT322404, MT293195, MT293192, MT326150, MT263391, MT259251, MT293208, MT326095, MT326088, MT293176, MT263403, MT259263, MT259228, MT358719, MT358684, MT345805, MT350250, MT326118, MT326048, MT322394, MT259260, MT358723, MT358667, MT326116, MT159716, MN996529, MT326067, MT326066, MT293212, MT263418, MT326164, MT326069, MT326063, MT293196, MT293177, MT246471, MT345809, MT276597, MT358647, MT263447, MT350249, MT293187, MT246475, MT345835, MT322412, MT293172, MT358747, MT345858, MT345816, MT326106, MT293213, MT293179, MT263463, MT251975, MT246476, MT322411, MT246469, MT358661, MT293161, MT259244, MT345865, MT345861, MT293225, MT259248, MT358657, MT345833, MT293198, MT259227, MT358735, MT358698, MT345867, MT322420, MT188340, MT358646, MT350241, MT345872, MT326140, MT293189, MT233526, MT345815, MT259271, MT358687, MT293186, MT326099, MT326068, MT263444, MT345878, MT326127, MT263432, MT358721, MT358662, MT326154, MT293216, MT293207, MT293178, MT263440, MT263417, MT259286, MT326055, MT322407, MT263419, MT350243, MT345886, MT345827, MT326137, MT050493, MT012098, MT358734, MT326030, MT326029, MT358703, MT345853, MT345836, MT259277, MT345885, MT263445, MT246470, MT358671, MT326119, MT326027, MT345825, MT263074, MT246487, MT358693, MT345828, MT039873, MT345811, MT322421, MT259257, MT358730, MT263410, MT259237, MT358700, MT358688, MT350246, MT326061, MT322396, MT293182, MT246489, MT358658, MT293171, MT263439, MT251974, MT246486, MN938384, MT358710, MT345830, MT326097, MT263411, MT251973, MT246490, MT345798, MT326042, MT044258, MT358739, MT263465, MT263443, MT263413, MT326086, MT326023, MT350242, MT345854, MT326162, MT263431, MT326089, MT263467, MT345849, MT326171, MT345802, MT358670, MT334537, MT326087, MT326056, MT263433, MT358733, MT345879, MT358738, MT358659, MT358655, MT350248, MT345846, MT358656, MT293222, MT293201, MT259275, MT293215, MT259246, MT358717, MT263425, MT240479, MT358653, MT293162, MT263402, MT246451, MT344951, MT344950, MT246457, MN996527, MT358690, MT226610

**Set 3** (n = 264)

MT350256, MT358650, MT358711, MT293211, MT358677, MT326166, MT163720, MT293184, MT246488, MT358682, MT263414, MT326081, MT326028, MT326104, MT326085, MT259243, MT322398, MT350238, MT322414, MT233523, MT198652, MT292573, MT233519, MT292574, MT292569, MT292575, MT326158, MT358713, MT326105, MT326138, MT345832, MT358709, MT259285, MT326149, MT326091, MT263448, MT263457, MT263400, MT326051, MT308702, MT358701, MT358654, MT263430, MT291832, MT259281, MT326098, MT358678, MT344952, MT327745, MT326120, MT263469, MT326113, MT300186, MT192765, MT358737, MT358668, MT322403, MT293209, MT251972, MT358732, MT345883, MT293165, MT358691, MT345826, MT263450, MT259264, MT358644, MT259267, MT358651, MT358648, MT358712, MT326128, MT326074, MT326070, MT358728, MT358665, MT326169, MT326078, MT263464, MT259261, MT258382, MT345870, MT263423, MT358660, MT326134, MT291833, MT358689, MT345881, MT345829, MT326076, MT263436, MT326075, MT263452, MT358746, MT320891, MT326082, MT263392, MT326189, MT293190, MT334536, MT072688, MT358729, MT358724, MT358699, MT358652, MT326172, MT293156, MT263420, MT326159, MT326040, MT358666, MT326133, MT291830, MT259256, MT358683, MT345817, MT326071, MT293200, MT358681, MT358664, MT358645, MT345874, MT293205, MT263405, MT345856, MT326058, MT326093, MT308703, MT358672, MT263424, MT326180, MT293224, MT020781, MT326148, MT263449, MT263415, MT246482, MT263422, MT246479, MT246464, MT259282, MT326084, MT291826, MT251980, MT251977, MT259249, MT263416, MT293173, MT263454, MT345859, MT246468, MT263412, MT259245, MT262993, MT259253, MT246453, MT345866, MT345880, MT345876, MT263442, MT263408, MT358680, MT326191, MT246449, MT358714, MT326125, MT326112, MT251979, MT326049, MT358697, MT358731, MT308704, MT358663, MT253696, MT077125, MT253706, MT326185, MT263434, MT326039, MT292572, MT291829, MT345855, MT246473, MT345801, MT326090, MT358673, MT326135, MT326130, MT246472, MT256924, MT259266, MT292570, MT358679, MT293188, MT326111, MT259252, MT188339, MT358692, MT188341, MT263456, MT345824, MT293175, MT263428, MT263462, MT334557, MT345844, MT326041, MT263383, MT259268, MT326160, MT326035, MT326046, MT259274, MT326036, MT326168, MT326147, MT259235, MT345812, MT326053, MT293164, MT263390, MT293169, MT326124, MT263441, MT326152, MT326123, MT345814, MT293170, MT293158, MT345847, MT345822, MT326184, MT345860, MT326151, MT326094, MT326177, MT293191, MT358720, MT358708, MT263382, MT326054, MT293167, MT246456, MT263388, MT326044, MT326031, MT246485, MT263386, MT345848, MT326174, MT263398, MT263384, MT326156, MT326034, MT326110, MT259276, MT358726, MT345823, MT358685, MT293223, MT326072, MT358704, MT334526, MT334561

TABLE S1B

LISITNG OF EBOLA VIRUSES SEQUENCES USED IN THE STUDY

**Old strains** (n = 57)

AF086833, KC242801, KY425630, KY425637, KY425639, KY425647, KY425649, KY425652, KY425656, MH121166, MH121168, KR063671, KC242791, KC242792, KR063672, JQ352763, KR867676, KU182898, KU182899, KU182900, KU182901, KU182902, KU182903, KU182904, KU182905, KU182906, KU182907, KU182908, KU182909, KY425636, KY425653, MG572235, KC242796, KC242799, KR824526, MH121165, KC242793, KC242794, KY785939, KY785940, KY785947, KY785948, KY785949, KY785965, KY785969, KY785970, KY786017, KC242800, KF113528, KC242784, KC242785, KC242786, KC242787, KC242788, KC242789, KC242790, HQ613403

**West Africa strains** (n = 1044)

KU296663, KX000398, KX000399, KP096420, KP096421, KP096422, KT013254, KT013257, KX000400, KY425633, KY425645, KY425648, KY425654, KY425657, MG572230, MG572231, MG572232, MG572233, MG572234, KR817187, KR817188, KR817189, KR817179, KR817180, KR817181, KR817182, KR817183, KR817184, KR817185, KR817186, KR817190, KR817191, KR817192, KR817193, KR817194, KR817195, KR817196, KR817197, KR817198, KR817199, KR817200, KR817201, KR817202, KR817203, KR817204, KR817205, KR817206, KR817207, KR817208, KR817209, KR817210, KR817211, KR817212, KR817213, KM034549, KM034550, KR817214, KR817215, KR817216, KM034551, KM034553, KM034554, KM034556, KM034557, KM034558, KM034559, KM034560, KM034561, KM034562, KR817217, KM233049, KR817218, KM233035, KM233036, KM233037, KM233038, KM233039, KM233040, KM233041, KM233042, KM233043, KM233044, KM233045, KM233053, KM233116, KR817219, KR817220, KM034555, KM233046, KM233047, KM233054, KM233055, KM233056, KM233048, KM233050, KM233051, KM233057, KM233058, KM233061, KM233062, KM233063, KM233065, KM233069, KM233071, KR817221, KR817222, KR817223, KR817224, KR817225, KR817226, KM233052, KM233064, KM233066, KM233067, KM233070, KM233072, KM233073, KM233074, KM233075, KM233076, KM233077, KM233079, KM233080, KM233081, KM233082, KM233084, KM233085, KM233086, KM233087, KM233089, KM233091, KM233092, KM233093, KM233095, KM233096, KM233097, KM233098, KM233100, KM233101, KM233102, KM233103, KR105200, KR817227, KR817228, KR817229, KM233088, KM233099, KM233104, KM233105, KM233106, KM233107, KM233109, KM233110, KM233112, KM233113, KM233115, KR105204, KR105205, KU156714, KU156715, KM233114, KR105206, KR105207, KR817230, KR817231, KR817232, KR817233, KR817234, KR817235, KR105209, KR105210, KR817236, KR817237, KR817238, KT725262, KJ660346, KJ660347, KJ660348, KM519951, KR074996, KR075001, KR075002, KR075003, KR817239, KR824525, KT765130, KT765131, KU143775, KU143776, KU143777, KU143778, KU143779, KU143780, KU143781, KU143782, KU143783, KU143784, KU143785, KU143786, KU143787, KU143788, KU143789, KU143790, KU143791, KU143792, KU143793, KU143794, KU143795, KU143796, KU143797, KU143798, KU143799, KU143800, KU143801, KU143802, KU143803, KU143804, KU143805, KU143806, KU143807, KU143808, KU143809, KU143810, KU143811, KU143812, KU143813, KU143814, KU143815, KU143816, KU143817, KU143818, KU143819, KU143820, KU143821, KU143822, KU143823, KU143824, KU143825, KU143826, KU143827, KU143828, KU143829, KU143830, KU143831, KU143832, KU143833, KU143834, KR105214, KR817240, KR817241, KT725388, KR105213, KR105215, KR105216, KR105217, KR105218, KR105219, KR105221, KR105222, KR105223, KR105224, KR105225, KR105226, KR105227, KR105228, KR105241, KR817242, KT725275, KR105229, KR105230, KR105231, KR105232, KR105233, KR105234, KR105235, KR105237, KR105238, KR105240, KR105242, KR105243, KR817243, KR817244, KR817245, KR105239, KR105244, KR105247, KR105248, KR817107, KR105249, KR105250, KR105251, KR105252, KR105256, KR105258, KR817108, KR817109, KR817110, KR817111, KR817112, KR817113, KT725324, KX013101, KR534507, KR534508, KR534509, KR534586, KR817114, KR817115, KR534510, KR105262, KR534511, KR817116, KR817117, KR817118, KR817119, KY558988, KP178538, KR105263, KR105264, KR105265, KR105266, KR105268, KR105269, KR105270, KR105271, KR817120, KR817121, KX013091, KX013092, KX013093, KX013094, KX013097, KX013098, KX013099, KR817122, KR817123, MH425138, KR105274, KR105275, KR105276, KR105277, KR105278, KR105279, KR105280, KR105281, KR105286, KR534512, KT725311, KU220277, KR105283, KR105284, KR105285, KR105287, KR105288, KR105289, KR105291, KR105292, KR105293, KR105294, KR105295, KR534513, KR534514, KR534515, KR817124, KR817125, KR817126, KR817127, KR817128, KR819004, KT725257, KT725259, KT725348, KT725363, KT725377, KT725380, KT725383, MK044559, KP271018, KP271020, KR105298, KR534516, KR534517, KR653252, KT725335, KT725337, KT725367, KT725387, KT725392, KY401659, KR105300, KR105301, KR534518, KR534519, KR653239, KR653241, KR653251, KR653267, KR817129, KR817130, KT725286, KT725331, KT725364, KU220278, KU220279, KU220280, MK044560, KP120616, KP184503, KR105303, KR105306, KR534520, KR534521, KR534522, KR534587, KR534588, KR653227, KR653265, KR653294, KT725256, KT725281, KT725283, KT725297, KT725341, KT725347, KT725349, KT725356, KT725369, KU220281, KU220282, KX009892, KX009893, KX009894, KX009895, MK044561, KR534523, KR653296, KR817131, KR817132, KR817133, KR817134, KR105307, KR653235, KR653246, KR653279, KR653287, KR817067, KR817068, KR817069, KR817070, KR817071, KT725289, KX009896, KX009897, KY558985, KY558987, KR105308, KR105311, KR653263, KR653278, KR817072, KR817073, KT587346, KT589389, KT725255, KT725260, KX009899, KX009900, KX009901, KR105312, KR105313, KR105315, KR534524, KR653286, KR817074, KT725263, KT725265, KT725317, KT725385, KY401660, KY401661, KP240931, KR105316, KR105317, KR105318, KR105322, KR534525, KR534526, KR653297, KR817075, KR817076, KR817077, KR817078, KT725296, KT725319, KT725352, KY401662, KR105320, KR105321, KR105323, KR105325, KR105326, KR105327, KR534577, KR653269, KR653280, KX009902, KY401663, KR105328, KR105329, KR105330, KR105331, KR534527, KR534528, KR534529, KR534530, KR534531, KR534532, KR534578, KR653244, KR653250, KR653291, KR653305, KR817079, KR817080, KT725295, KT725308, KT725325, KT725353, KT725362, KT725371, KT725379, KP240932, KP759741, KP759747, KP759755, KR105332, KR105333, KR105335, KR105338, KR105339, KR105340, KR105341, KR105342, KR105343, KR105344, KR534534, KR534535, KR534536, KR534579, KR534580, KR534581, KR653229, KR653232, KR653261, KR653266, KR653284, KR653300, KR653303, KT587345, KT725276, KP759663, KP759668, KP759688, KP759718, KP759734, KP759740, KP759756, KR105345, KR105346, KR105349, KR534537, KR534582, KR653260, KR653288, KR817081, KR817082, KR817083, KP759608, KP759615, KP759618, KP759666, KP759670, KP759678, KP759683, KP759691, KP759692, KP759694, KP759742, KR534538, KR534539, KR534540, KR534583, KR534584, KR653230, KR653273, KR653289, KR817084, KR817085, KR817086, KR817087, KT725258, KT725268, KT725269, KT725270, KT725310, KT725368, KY401664, KY401665, KP240933, KP759710, KP759711, KP759713, KP759714, KP759715, KP759716, KP759717, KP759719, KP759721, KP759722, KP759723, KR534541, KR534543, KR534544, KR534545, KR534546, KR534548, KR534549, KR534585, KR534589, KR534590, KR534591, KR653225, KR653233, KR817088, KR817089, KY401667, KP759720, KP759724, KP759725, KP759727, KP759728, KP759730, KP759731, KP759732, KP759733, KP759735, KP759736, KP759737, KR534550, KR534552, KR534553, KR534554, KR534555, KR534556, KR653283, KR653293, KR817135, KR817136, KR817137, KR817138, KR817139, KX009904, KX009905, KP759738, KP759739, KR534557, KR534558, KR534559, KR534560, KR653234, KR653262, KR653274, KT633510, KT725287, KT725375, KT725391, KP342330, KP759743, KP759744, KP759745, KP759746, KP759748, KP759750, KP759751, KP759752, KR534561, KR534562, KR534564, KR534565, KR534575, KR653226, KR653254, KR817141, KR817142, KR817143, KR534563, KR534566, KR653255, KR817144, KP260799, KP759758, KP759759, KP759760, KR534568, KR534569, KR534570, KR534571, KR534572, KR534573, KR534574, KR653247, KR653268, KR653275, KR653285, KR653301, KR653302, KR817145, KR817146, KR817147, KX009906, KY401674, KY401675, MG948587, KP759655, KP759659, KP759669, KP759671, KP759761, KP759764, KP759765, KP759766, KP759767, KP759768, KR653272, KR653277, KR653282, KR653298, KR817148, KR817149, KR817150, KY401672, KP759595, KP759598, KP759599, KP759603, KP759660, KP759661, KP759662, KP759664, KP759665, KP759667, KP759672, KP759674, KP759675, KP759676, KP759677, KP759681, KP759682, KP759684, KP759685, KP759687, KR653228, KR653245, KR653248, KR653258, KR653304, KR817151, KR817152, KY558986, KP759600, KP759605, KP759673, KP759679, KP759689, KP759690, KR653236, KR653253, KR653256, KR653257, KR653292, KR817153, KX121419, KX121420, MG948588, KP759610, KP759614, KP759695, KP759696, KP759697, KP759698, KP759699, KP759700, KP759701, KP759702, KP759703, KP759704, KP759706, KR006947, KR653238, KR653240, KR653242, KR653264, KR817155, KR817156, KT725274, KT725303, MG948589, KP260800, KP260802, KP759705, KP759707, KP759709, KR653270, KR653271, KT725254, MG948590, MG948591, KR653231, KR653243, KR653259, KR653299, KR817157, KR817158, KR817159, KT725279, KT725370, KT725393, KY401668, KT725333, KX009907, KP260801, KP728283, KX009909, KR653237, KR653295, KR817161, KT725342, KX009910, KX009911, KT725290, KR817162, KR817163, KR817164, KR817165, KR817166, KY558984, KR817167, KR817168, KR817169, KR817170, KY401673, KR817171, KR817172, KR817173, KR817174, KY401669, KR817091, KR817175, KR817176, KR653224, KR817090, KR817092, KT725288, KR817093, KR817094, KU296835, KX121421, KP658432, KR817095, KY401670, KR817096, KR817097, KR817098, KR817099, KR817100, KR817101, KR817102, KT357827, KT357828, KT357830, KU220284, KT357831, KT357832, KT357833, KT357834, KT357835, KT357836, KT357837, KT357838, KT357839, KY401671, KR817103, KR817104, KR817105, KT357841, KT357842, KT357843, KT357844, KR817106, KU296773, KT357848, KT357849, KT725384, KU296549, KT357850, LC152433, KT357813, KT357852, KT357853, KT357814, KT357815, KT357816, KT357855, KT357817, KU296305, KT357818, KT357820, KT357821, KR025228, KT357822, KU296527, KU296766, KY366413, KY366414, KY366415, KY366417, KY366418, KY366419, KY366420, MF599506, MH470382, KU296764, MF599519, MF599507, KT357823, MF599522, MF599508, MF599509, KY366416, MF102255, KX121424, MF599510, MF599511, MF599505, MF599504, KT961624, MF599514, KU296819, MF599516, MF599518, KU296617, MF599517, KY401650, KY401654, KU296428, KY401651, KY401655, MH470375, MH470376, MH470377, MH470378, MH470379, KR074997, KR074998, KR074999, KR075000, KU220269, KU220270, KY401656, KY426684, KY426685, KY426686, KY426687, KY426688, KY426689, KY426690, KY426691, KY426692, KY426693, KY426694, KY426695, KY426696, KY426697, KY426698, KY426699, KY426700, KY426701, KY426702, KY426703, KY426704, KY426705, KY426706, KY426707, KY426708, KY426709, KY426710, KY426711, KY426713, KY426714, KY426715, KY426716, KY426717, KY426718, KY426719, KY426720, KY426721, KY426722, KY426723, KY426724, KY426725, KY426726, KY426727, KY426728, KY426729, KY426730, KY426731, KY426732, KY426733, KY471090, KY471092, KY471095, KY471122, KY471125, KU220271, KU220272, KY401652, MH470373, MH470374, KU220273, KT357859, KU296370, KY805810, KU220276, KY401653, KU296319, MH470380, MH470381, KY401643, KY401644, KY401645, KU052669, KU052670, KY744597, KY744596

**Congo** (n = 69)

MH733477, MH733478, MH898466, MH733479, MH733480, MH733481, MH733482, MH733483, MH733484, MH733485, MH733486, MH733487, MH733488, MH733489, MH733490, MH733491, MK007329, MK007330, MK163644, MK163645, MK163646, MK163647, MK163648, MK163649, MK163650, MK007331, MK007332, MK007333, MK007334, MK007335, MK007336, MK163651, MK163652, MK163653, MK163654, MK163655, MK163656, MK163658, MK163660, MK731985, MK731987, MK731988, MK731993, MK007337, MK007338, MK007339, MK007340, MK007341, MK007342, MK007343, MK007344, MK731986, MK731989, MK731990, MK731994, MK163661, MK731992, MK163662, MK163663, MK163665, MK163666, MK163667, MK163668, MK163669, MK163670, MK163672, MK163673, MK163674, MK163675
