# Supplementary material for: Rampant C→U Hypermutation in the Genomes of SARS-CoV-2 and Other Coronaviruses: Causes and Consequences for Their Short- and Long-Term Evolutionary Trajectories
Source: mSphere. 2020 Jun 24;5(3):e00408-20. doi: 10.1128/mSphere.00408-20 (PMC7316492; doi:10.1128/mSphere.00408-20)
Supplement: FIG S1 [file mSphere.00408-20-sf001.docx]

SUPPLEMENTARY DATA

FIGURE S1

FREQUENCIES OF TRANSITIONS IN ALL 5’ AND 3’ BASE CONTEXT COMBINATIONS


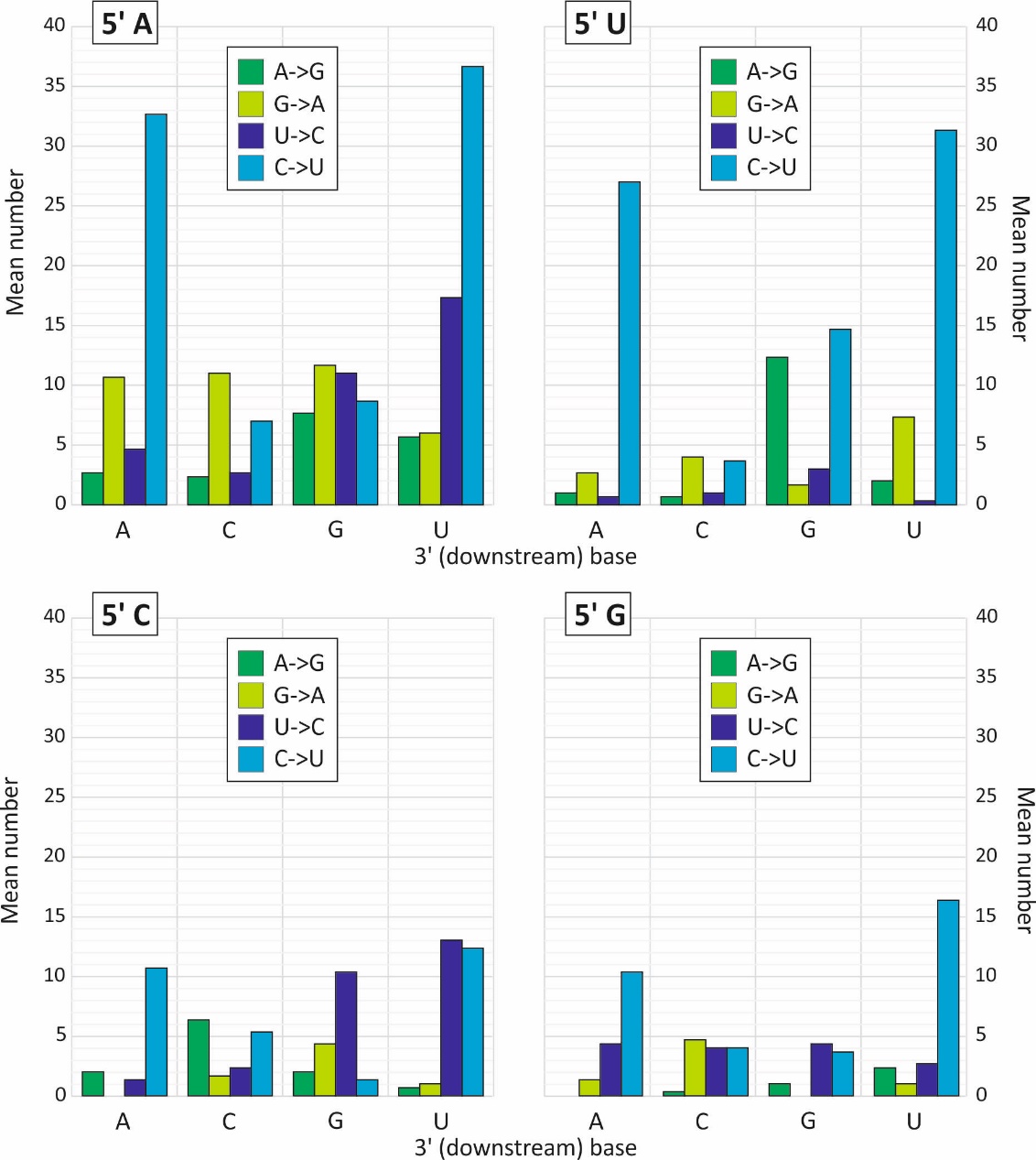


Effects of 5’ and 3’ bases on transition frequencies in SARS-CoV-2 full genome sequences.
