# Supplementary material for: Rampant C→U Hypermutation in the Genomes of SARS-CoV-2 and Other Coronaviruses: Causes and Consequences for Their Short- and Long-Term Evolutionary Trajectories
Source: mSphere. 2020 Jun 24;5(3):e00408-20. doi: 10.1128/mSphere.00408-20 (PMC7316492; doi:10.1128/mSphere.00408-20)
Supplement: TABLE S2 [file mSphere.00408-20-st002.docx]

SUPPLEMENTARY DATA

TABLE S2

SEQUENCES USED FOR COMPARISON OF CpG SUPPRESSION IN CORONAVIRUSES

| **Group** | **Virus** | **Accession No** | **G+C** | ***Ratio_*CpG** |
| --- | --- | --- | --- | --- |
| Sarbecovirus | SARS-CoV-2 | MN988713 | 38.01% | 0.4058 |
| Sarbecovirus | Bat SARS-like | MN996532 | 38.06% | 0.4086 |
| Sarbecovirus | SARS-CoV-1 | FJ882953 | 40.86% | 0.4675 |
| Sarbecovirus | Bat SARS-like | KF294457 | 40.83% | 0.4804 |
| Sarbecovirus | Bat SARS-like | KJ473813 | 40.98% | 0.4890 |
| Sarbecovirus | Bat SARS-like | KP886809 | 40.76% | 0.4788 |
| Sarbecovirus | Bat SARS-like | KJ473814 | 41.44% | 0.4844 |
| Sarbecovirus | Bat SARS-like | MG772934 | 38.85% | 0.4479 |
| Sarbecovirus | Bat SARS-like | KY352407 | 39.23% | 0.4590 |
| Sarbecovirus | Bat SARS-like | GU190215 | 40.48% | 0.5118 |
| Sarbecovirus | Bat SARS-like | JX993988 | 40.87% | 0.4833 |
| Sarbecovirus | Bat SARS-like | MK211374 | 40.88% | 0.4906 |
|  |  |  |  |  |
| Coronaviridae | Alphacoronavirus | KF430219 | 40.83% | 0.5065 |
| Coronaviridae | Alphacoronavirus | JQ989270 | 38.52% | 0.4956 |
| Coronaviridae | Alphacoronavirus | KJ473807 | 38.27% | 0.5270 |
| Coronaviridae | Alphacoronavirus | AF304460 | 38.26% | 0.4965 |
| Coronaviridae | Alphacoronavirus | KF294380 | 40.25% | 0.5938 |
| Coronaviridae | Alphacoronavirus | LC119077 | 38.83% | 0.5434 |
| Coronaviridae | Alphacoronavirus | HM245925 | 37.47% | 0.5314 |
| Coronaviridae | Alphacoronavirus | EU420138 | 38.14% | 0.4717 |
| Coronaviridae | Alphacoronavirus | EU420139 | 41.79% | 0.5954 |
| Coronaviridae | Alphacoronavirus | KJ473806 | 40.99% | 0.5717 |
| Coronaviridae | Alphacoronavirus | KJ473809 | 41.82% | 0.5729 |
| Coronaviridae | Alphacoronavirus | AF353511 | 42.02% | 0.5586 |
| Coronaviridae | Alphacoronavirus | DQ648858 | 40.12% | 0.5093 |
| Coronaviridae | Alphacoronavirus | EF203064 | 39.28% | 0.5203 |
| Coronaviridae | Alphacoronavirus | AY567487 | 34.46% | 0.4168 |
| Coronaviridae | Alphacoronavirus | KY073745 | 42.78% | 0.6223 |
| Coronaviridae | Alphacoronavirus | AJ271965 | 37.60% | 0.4538 |
| Coronaviridae | Betacoronavirus | AY585228 | 36.79% | 0.4812 |
| Coronaviridae | Betacoronavirus | KM349742 | 40.07% | 0.5491 |
| Coronaviridae | Betacoronavirus | AY597011 | 32.06% | 0.4584 |
| Coronaviridae | Betacoronavirus | AY700211 | 41.81% | 0.5971 |
| Coronaviridae | Betacoronavirus | KF636752 | 41.28% | 0.5891 |
| Coronaviridae | Betacoronavirus | KC545383 | 37.48% | 0.4740 |
| Coronaviridae | Betacoronavirus | JX869059 | 41.24% | 0.5554 |
| Coronaviridae | Betacoronavirus | EF065509 | 43.19% | 0.6319 |
| Coronaviridae | Betacoronavirus | EF065505 | 37.82% | 0.5115 |
| Coronaviridae | Betacoronavirus | KU762338 | 45.30% | 0.6260 |
| Coronaviridae | Betacoronavirus | EF065513 | 41.05% | 0.6711 |
| Coronaviridae | Betacoronavirus | AY274119 | 40.76% | 0.4598 |
| Coronaviridae | Deltacoronavirus | JQ065048 | 39.36% | 0.5233 |
| Coronaviridae | Deltacoronavirus | FJ376619 | 38.69% | 0.4380 |
| Coronaviridae | Deltacoronavirus | JQ065043 | 43.19% | 0.5557 |
| Coronaviridae | Deltacoronavirus | FJ376622 | 42.51% | 0.5611 |
| Coronaviridae | Deltacoronavirus | JQ065044 | 39.83% | 0.4536 |
| Coronaviridae | Deltacoronavirus | JQ065047 | 38.13% | 0.4131 |
| Coronaviridae | Deltacoronavirus | JQ065049 | 35.06% | 0.4270 |
| Coronaviridae | Deltacoronavirus | EU111742 | 39.24% | 0.5097 |
| Coronaviridae | Gammacoronavirus | IBACGB | 37.93% | 0.5009 |
|  |  |  |  |  |
| Arteriviridae | Arteriviridae | MF324848 | 51.89% | 0.7836 |
| Arteriviridae | Arteriviridae | X53459 | 51.66% | 0.7504 |
| Arteriviridae | Arteriviridae | KP026921 | 52.13% | 0.6931 |
| Arteriviridae | Arteriviridae | AF180391 | 50.09% | 0.6844 |
| Arteriviridae | Arteriviridae | KR139839 | 52.40% | 0.7636 |
| Arteriviridae | Arteriviridae | KM677927 | 51.95% | 0.7435 |
| Arteriviridae | Arteriviridae | KR862307 | 51.60% | 0.7170 |
| Arteriviridae | Arteriviridae | KT166441 | 51.25% | 0.6914 |
| Arteriviridae | Arteriviridae | KC787658 | 48.63% | 0.6703 |
| Arteriviridae | Arteriviridae | KP126831 | 54.22% | 0.7683 |
| Arteriviridae | Arteriviridae | JX473849 | 50.21% | 0.6896 |
| Arteriviridae | Arteriviridae | KT447550 | 50.84% | 0.6872 |
| Arteriviridae | Arteriviridae | KM110938 | 50.90% | 0.6661 |
| Arteriviridae | Arteriviridae | KC787630 | 52.16% | 0.8098 |
| Arteriviridae | Arteriviridae | PRU87392 | 52.84% | 0.7048 |
| Arteriviridae | Arteriviridae | KP280006 | 52.51% | 0.6543 |
| Arteriviridae | Arteriviridae | KU302440 | 53.75% | 0.7206 |
| Arteriviridae | Arteriviridae | M96262 | 52.62% | 0.6990 |
| Arteriviridae | Arteriviridae | LDU15146 | 49.43% | 0.6095 |
| Arteriviridae | Arteriviridae | JN116253 | 50.26% | 0.7997 |
| RNA_virus | Arenaviridae | NC_001803 | 33.00% | 0.23 |
| RNA_virus | Arenaviridae | NC_001925 | 33.30% | 0.243 |
| RNA_virus | Arenaviridae | NC_001652 | 33.70% | 0.672 |
| RNA_virus | Arenaviridae | NC_004108 | 35.00% | 0.304 |
| RNA_virus | Arenaviridae | FLCP3A | 35.80% | 0.235 |
| RNA_virus | Arenaviridae | FLCPB1A | 35.80% | 0.218 |
| RNA_virus | Arenaviridae | AF371337 | 36.10% | 0.244 |
| RNA_virus | Arenaviridae | NC_003461 | 36.30% | 0.395 |
| RNA_virus | Astroviridae | NC_002728 | 37.10% | 0.439 |
| RNA_virus | Astroviridae | NC_003045 | 37.10% | 0.481 |
| RNA_virus | Astroviridae | AF288297 | 37.30% | 0.307 |
| RNA_virus | Bunyaviridae | HPSVRPLA | 37.50% | 0.395 |
| RNA_virus | Bunyaviridae | NC_002306 | 37.60% | 0.454 |
| RNA_virus | Bunyaviridae | NC_002199 | 37.70% | 0.399 |
| RNA_virus | Bunyaviridae | NC_001489 | 37.90% | 0.149 |
| RNA_virus | Bunyaviridae | NC_001795 | 38.00% | 0.309 |
| RNA_virus | Bunyaviridae | NC_001906 | 38.10% | 0.457 |
| RNA_virus | Bunyaviridae | NC_002645 | 38.30% | 0.495 |
| RNA_virus | Bunyaviridae | NC_001608 | 38.40% | 0.515 |
| RNA_virus | Bunyaviridae | NC_003996 | 38.40% | 0.221 |
| RNA_virus | Bunyaviridae | NC_004109 | 38.40% | 0.278 |
| RNA_virus | Bunyaviridae | SHVAGM27 | 38.40% | 0.15 |
| RNA_virus | Bunyaviridae | NC_001736 | 38.50% | 0.311 |
| RNA_virus | Bunyaviridae | NC_001752 | 38.50% | 0.208 |
| RNA_virus | Bunyaviridae | NC_002205 | 38.50% | 0.289 |
| RNA_virus | Bunyaviridae | HPSNUPR | 38.60% | 0.367 |
| RNA_virus | Bunyaviridae | TUVM5302 | 38.60% | 0.16 |
| RNA_virus | Bunyaviridae | NC_001450 | 38.70% | 0.274 |
| RNA_virus | Bunyaviridae | NC_001871 | 38.80% | 0.333 |
| RNA_virus | Bunyaviridae | NC_001364 | 38.90% | 0.337 |
| RNA_virus | Caliciviridae | NC_001617 | 39.00% | 0.25 |
| RNA_virus | Caliciviridae | NC_002206 | 39.10% | 0.261 |
| RNA_virus | Caliciviridae | NC_004159 | 39.10% | 0.226 |
| RNA_virus | Caliciviridae | NC_002204 | 39.20% | 0.393 |
| RNA_virus | Caliciviridae | HANC12GP | 39.30% | 0.187 |
| RNA_virus | Caliciviridae | AF288298 | 39.40% | 0.276 |
| RNA_virus | Caliciviridae | NC_001897 | 39.50% | 0.235 |
| RNA_virus | Caliciviridae | NC_002201 | 39.80% | 0.412 |
| RNA_virus | Caliciviridae | HPSGPGO | 39.90% | 0.301 |
| RNA_virus | Caliciviridae | NC_003467 | 40.10% | 0.211 |
| RNA_virus | Caliciviridae | NC_004291 | 40.40% | 0.267 |
| RNA_virus | Caliciviridae | NC_001490 | 40.60% | 0.297 |
| RNA_virus | Caliciviridae | NC_001511 | 40.60% | 0.374 |
| RNA_virus | Caliciviridae | NC_004161 | 40.60% | 0.557 |
| RNA_virus | Caliciviridae | NC_004292 | 40.80% | 0.315 |
| RNA_virus | Caliciviridae | NC_001463 | 41.10% | 0.312 |
| RNA_virus | Caliciviridae | NC_004297 | 41.20% | 0.267 |
| RNA_virus | Caliciviridae | NC_001867 | 41.40% | 0.631 |
| RNA_virus | Caliciviridae | NC_001452 | 41.50% | 0.391 |
| RNA_virus | Caliciviridae | NC_003044 | 41.60% | 0.493 |
| RNA_virus | Caliciviridae | NC_004158 | 41.70% | 0.293 |
| RNA_virus | Caliciviridae | NC_001846 | 41.80% | 0.597 |
| RNA_virus | Caliciviridae | NC_002526 | 41.80% | 0.348 |
| RNA_virus | Caliciviridae | NC_002022 | 41.90% | 0.381 |
| RNA_virus | Caliciviridae | NC_003436 | 42.00% | 0.559 |
| RNA_virus | Caliciviridae | NC_002021 | 42.10% | 0.398 |
| RNA_virus | Caliciviridae | HIVMNCG | 42.30% | 0.233 |
| RNA_virus | Caliciviridae | NC_001921 | 42.40% | 0.493 |
| RNA_virus | Caliciviridae | NC_003976 | 42.60% | 0.303 |
| RNA_virus | Caliciviridae | AF485264 | 42.70% | 0.219 |
| RNA_virus | Caliciviridae | NC_001430 | 42.80% | 0.281 |
| RNA_virus | Caliciviridae | NC_001550 | 43.00% | 0.462 |
| RNA_virus | Caliciviridae | NC_003988 | 43.00% | 0.501 |
| RNA_virus | Caliciviridae | NC_003675 | 43.20% | 0.349 |
| RNA_virus | Caliciviridae | AY129248 | 43.40% | 0.291 |
| RNA_virus | Caliciviridae | NC_001484 | 43.40% | 0.444 |
| RNA_virus | Caliciviridae | NC_002470 | 43.50% | 0.42 |
| RNA_virus | Caliciviridae | AF296091 | 43.70% | 0.322 |
| RNA_virus | Caliciviridae | NC_001503 | 43.70% | 0.453 |
| RNA_virus | Caliciviridae | NC_002023 | 43.70% | 0.478 |
| RNA_virus | Caliciviridae | NC_004293 | 43.90% | 0.326 |
| RNA_virus | Filoviridae | AF296115 | 44.30% | 0.317 |
| RNA_virus | Filoviridae | AF296119 | 44.30% | 0.367 |
| RNA_virus | Flaviviridae | AF296093 | 44.40% | 0.356 |
| RNA_virus | Flaviviridae | NC_004107 | 44.50% | 0.517 |
| RNA_virus | Flaviviridae | AF296087 | 44.60% | 0.367 |
| RNA_virus | Flaviviridae | FCU13992 | 44.80% | 0.565 |
| RNA_virus | Flaviviridae | NC_003985 | 44.80% | 0.345 |
| RNA_virus | Flaviviridae | NC_001943 | 44.90% | 0.438 |
| RNA_virus | Flaviviridae | AB038528 | 45.00% | 0.354 |
| RNA_virus | Flaviviridae | NC_001413 | 45.00% | 0.402 |
| RNA_virus | Flaviviridae | NC_000855 | 45.10% | 0.544 |
| RNA_virus | Flaviviridae | AF231769 | 45.20% | 0.359 |
| RNA_virus | Flaviviridae | NC_001428 | 45.20% | 0.448 |
| RNA_virus | Flaviviridae | NC_001552 | 45.40% | 0.496 |
| RNA_virus | Flaviviridae | NC_001831 | 45.40% | 0.43 |
| RNA_virus | Flaviviridae | AF037405 | 45.50% | 0.412 |
| RNA_virus | Flaviviridae | BVU18059 | 45.50% | 0.371 |
| RNA_virus | Flaviviridae | NC_003635 | 45.50% | 0.306 |
| RNA_virus | Flaviviridae | NC_001722 | 45.70% | 0.318 |
| RNA_virus | Flaviviridae | NC_001719 | 45.80% | 0.447 |
| RNA_virus | Flaviviridae | BVDCG | 45.80% | 0.41 |
| RNA_virus | Flaviviridae | AB070225 | 45.90% | 0.694 |
| RNA_virus | Flaviviridae | NC_003677 | 45.90% | 0.417 |
| RNA_virus | Flaviviridae | AF479590 | 46.00% | 0.573 |
| RNA_virus | Flaviviridae | NC_001474 | 46.10% | 0.431 |
| RNA_virus | Flaviviridae | NC_001481 | 46.10% | 0.597 |
| RNA_virus | Flaviviridae | NC_004294 | 46.20% | 0.318 |
| RNA_virus | Flaviviridae | NC_002058 | 46.30% | 0.538 |
| RNA_virus | Flaviviridae | NC_003678 | 46.30% | 0.384 |
| RNA_virus | Flaviviridae | NC_003982 | 46.40% | 0.541 |
| RNA_virus | Flaviviridae | NC_001654 | 46.40% | 0.428 |
| RNA_virus | Flaviviridae | FCLF4 | 46.70% | 0.568 |
| RNA_virus | Flaviviridae | NC_001475 | 46.70% | 0.414 |
| RNA_virus | Flaviviridae | NC_001477 | 46.70% | 0.452 |
| RNA_virus | Flaviviridae | PTU90951 | 46.70% | 0.433 |
| RNA_virus | Flaviviridae | NC_001498 | 46.90% | 0.495 |
| RNA_virus | Flaviviridae | POL3L37 | 46.90% | 0.538 |
| RNA_virus | Flaviviridae | NC_002640 | 47.10% | 0.384 |
| RNA_virus | Flaviviridae | NC_001472 | 47.20% | 0.552 |
| RNA_virus | Flaviviridae | ERVPOLY | 47.30% | 0.561 |
| RNA_virus | Flaviviridae | AB042808 | 47.40% | 0.382 |
| RNA_virus | Flaviviridae | AF091736 | 47.70% | 0.662 |
| RNA_virus | Flaviviridae | AB039774 | 47.80% | 0.413 |
| RNA_virus | Hepadnaviridae | SMU15301 | 47.80% | 0.694 |
| RNA_virus | Hepadnaviridae | NC_002551 | 47.90% | 0.669 |
| RNA_virus | Hepadnaviridae | NC_001612 | 48.00% | 0.502 |
| RNA_virus | Hepadnaviridae | NC_001959 | 48.00% | 0.467 |
| RNA_virus | Hepadnaviridae | NC_003986 | 48.00% | 0.556 |
| RNA_virus | Hepadnaviridae | NC_001924 | 48.10% | 0.752 |
| RNA_virus | Hepadnaviridae | AF321298 | 48.20% | 0.679 |
| RNA_virus | Hepadnaviridae | NC_004542 | 48.20% | 0.678 |
| RNA_virus | Hepatitis E-like viruses | MNGPOLY | 48.30% | 0.562 |
| RNA_virus | Hepatitis E-like viruses | AF242585 | 48.40% | 0.541 |
| RNA_virus | Hepatitis E-like viruses | NC_001512 | 48.40% | 0.761 |
| RNA_virus | Hepatitis E-like viruses | NC_003676 | 48.40% | 0.411 |
| RNA_virus | Hepatitis E-like viruses | NC_001786 | 48.50% | 0.76 |
| RNA_virus | Hepatitis E-like viruses | AB039781 | 48.60% | 0.44 |
| RNA_virus | Orthomyxoviridae | AB039782 | 48.60% | 0.431 |
| RNA_virus | Orthomyxoviridae | NC_000943 | 48.70% | 0.514 |
| RNA_virus | Orthomyxoviridae | NC_002534 | 48.70% | 0.591 |
| RNA_virus | Orthomyxoviridae | NC_003899 | 48.70% | 0.81 |
| RNA_virus | Orthomyxoviridae | AF093797 | 48.80% | 0.411 |
| RNA_virus | Orthomyxoviridae | NC_002469 | 48.80% | 0.509 |
| RNA_virus | Orthomyxoviridae | NC_003983 | 48.80% | 0.68 |
| RNA_virus | Orthomyxoviridae | SOUCAPPRO | 49.00% | 0.466 |
| RNA_virus | Orthomyxoviridae | AB039777 | 49.10% | 0.451 |
| RNA_virus | Paramyxovirus | NC_001366 | 49.20% | 0.653 |
| RNA_virus | Paramyxovirus | NC_003908 | 49.20% | 0.853 |
| RNA_virus | Paramyxovirus | NC_001859 | 49.30% | 0.59 |
| RNA_virus | Paramyxovirus | NC_001896 | 49.30% | 0.43 |
| RNA_virus | Paramyxovirus | NC_002168 | 49.30% | 0.543 |
| RNA_virus | Paramyxovirus | NC_002615 | 49.30% | 0.611 |
| RNA_virus | Paramyxovirus | NC_003977 | 49.30% | 0.523 |
| RNA_virus | Paramyxovirus | SVDMPS | 49.40% | 0.609 |
| RNA_virus | Paramyxovirus | LDU15146 | 49.50% | 0.609 |
| RNA_virus | Paramyxovirus | NC_001479 | 49.50% | 0.514 |
| RNA_virus | Picornaviridae | NC_001514 | 49.50% | 0.485 |
| RNA_virus | Picornaviridae | AB039780 | 49.60% | 0.449 |
| RNA_virus | Picornaviridae | AB081723 | 49.70% | 0.431 |
| RNA_virus | Picornaviridae | HCU07611 | 49.70% | 0.459 |
| RNA_virus | Picornaviridae | NC_002031 | 49.70% | 0.383 |
| RNA_virus | Picornaviridae | AY134748 | 49.80% | 0.434 |
| RNA_virus | Picornaviridae | HBV131568 | 49.90% | 0.62 |
| RNA_virus | Picornaviridae | AF258618 | 50.00% | 0.643 |
| RNA_virus | Picornaviridae | AY032605 | 50.00% | 0.451 |
| RNA_virus | Picornaviridae | AB039779 | 50.10% | 0.425 |
| RNA_virus | Picornaviridae | AF145896 | 50.10% | 0.448 |
| RNA_virus | Picornaviridae | CRNAORFS | 50.10% | 0.446 |
| RNA_virus | Picornaviridae | NC_001449 | 50.10% | 0.759 |
| RNA_virus | Picornaviridae | NC_001543 | 50.10% | 0.588 |
| RNA_virus | Picornaviridae | NC_003092 | 50.10% | 0.684 |
| RNA_virus | Picornaviridae | AB039775 | 50.30% | 0.455 |
| RNA_virus | Picornaviridae | RHU54983 | 50.30% | 0.611 |
| RNA_virus | Picornaviridae | NC_003077 | 50.40% | 0.696 |
| RNA_virus | Picornaviridae | KUNCG | 50.60% | 0.536 |
| RNA_virus | Picornaviridae | NC_001655 | 50.60% | 0.592 |
| RNA_virus | Picornaviridae | RHDVCGS | 50.60% | 0.609 |
| RNA_virus | Picornaviridae | HECGENRA | 50.70% | 0.531 |
| RNA_virus | Picornaviridae | NC_001940 | 50.80% | 0.535 |
| RNA_virus | Picornaviridae | AF295785 | 51.00% | 0.601 |
| RNA_virus | Picornaviridae | NC_001547 | 51.00% | 0.9 |
| RNA_virus | Picornaviridae | NC_001560 | 51.00% | 0.48 |
| RNA_virus | Picornaviridae | NC_001563 | 51.10% | 0.579 |
| RNA_virus | Picornaviridae | NC_001564 | 51.30% | 0.798 |
| RNA_virus | Picornaviridae | NC_001542 | 51.40% | 0.473 |
| RNA_virus | Picornaviridae | NC_001544 | 51.40% | 0.819 |
| RNA_virus | Picornaviridae | NC_001437 | 51.50% | 0.584 |
| RNA_virus | Picornaviridae | NC_002532 | 51.60% | 0.75 |
| RNA_virus | Picornaviridae | SINOCK82 | 51.70% | 0.915 |
| RNA_virus | Picornaviridae | HCA249939 | 52.20% | 0.644 |
| RNA_virus | Picornaviridae | FMDVALF | 52.30% | 0.8 |
| RNA_virus | Picornaviridae | NC_001885 | 52.30% | 0.526 |
| RNA_virus | Picornaviridae | AY150312 | 52.40% | 0.708 |
| RNA_virus | Picornaviridae | NC_003218 | 52.70% | 0.524 |
| RNA_virus | Picornaviridae | NC_001961 | 52.90% | 0.696 |
| RNA_virus | Picornaviridae | NC_001501 | 53.00% | 0.505 |
| RNA_virus | Retroviridae | NC_001819 | 53.20% | 0.515 |
| RNA_virus | Retroviridae | NC_003215 | 53.20% | 0.892 |
| RNA_virus | Retroviridae | NC_003323 | 53.20% | 0.539 |
| RNA_virus | Retroviridae | NC_003992 | 53.20% | 0.853 |
| RNA_virus | Retroviridae | NC_003687 | 53.30% | 0.515 |
| RNA_virus | Retroviridae | NC_001362 | 53.40% | 0.512 |
| RNA_virus | Retroviridae | NC_000940 | 53.50% | 0.52 |
| RNA_virus | Retroviridae | NC_001436 | 53.50% | 0.58 |
| RNA_virus | Retroviridae | NC_001672 | 53.70% | 0.551 |
| RNA_virus | Retroviridae | NC_000858 | 53.80% | 0.616 |
| RNA_virus | Retroviridae | NC_001488 | 53.80% | 0.564 |
| RNA_virus | Retroviridae | NC_001500 | 53.80% | 0.572 |
| RNA_virus | Retroviridae | NC_001702 | 53.90% | 0.53 |
| RNA_virus | Retroviridae | AF331718 | 54.00% | 0.538 |
| RNA_virus | Retroviridae | NC_001363 | 54.10% | 0.587 |
| RNA_virus | Retroviridae | NC_001407 | 54.10% | 0.681 |
| RNA_virus | Retroviridae | NC_001502 | 54.10% | 0.587 |
| RNA_virus | Retroviridae | NC_002554 | 54.10% | 0.854 |
| RNA_virus | Retroviridae | NC_001414 | 54.10% | 0.636 |
| RNA_virus | Retroviridae | NC_003690 | 54.30% | 0.491 |
| RNA_virus | Retroviridae | NC_004421 | 54.60% | 0.722 |
| RNA_virus | Retroviridae | AB074917 | 54.70% | 0.794 |
| RNA_virus | Retroviridae | NC_001815 | 54.80% | 0.573 |
| RNA_virus | Retroviridae | AB074915 | 54.90% | 0.821 |
| RNA_virus | Retroviridae | NC_001809 | 54.90% | 0.561 |
| RNA_virus | Retroviridae | NC_002527 | 55.00% | 0.944 |
| RNA_virus | Retroviridae | AF154271 | 55.20% | 0.932 |
| RNA_virus | Retroviridae | NC_001499 | 55.20% | 0.417 |
| RNA_virus | Retroviridae | AY115488 | 55.30% | 0.766 |
| RNA_virus | Retroviridae | NC_004004 | 55.30% | 0.949 |
| RNA_virus | Retroviridae | AB074920 | 55.40% | 0.801 |
| RNA_virus | Retroviridae | AB074918 | 55.50% | 0.769 |
| RNA_virus | Retroviridae | HPCEGS | 55.70% | 0.723 |
| RNA_virus | Retroviridae | BCA011099 | 55.80% | 0.652 |
| RNA_virus | Retroviridae | HCV12083 | 55.90% | 0.708 |
| RNA_virus | Retroviridae | HCV4APOLY | 56.20% | 0.754 |
| RNA_virus | Rhabdovirus | NC_004064 | 56.60% | 0.726 |
| RNA_virus | Rhabdovirus | AY228235 | 56.80% | 0.627 |
| RNA_virus | Rhabdovirus | AF177036 | 56.90% | 0.674 |
| RNA_virus | Rhabdovirus | HCV1480 | 57.10% | 0.716 |
| RNA_virus | Rhabdovirus | pT7MNV3-2 | 57.30% | 0.6331 |
| RNA_virus | Rhabdovirus | HGU22303 | 57.80% | 0.693 |
| RNA_virus | Togavirus | NC_001434 | 57.90% | 0.817 |
| RNA_virus | Togavirus | NC_001837 | 57.90% | 0.652 |
| RNA_virus | Togavirus | AF011751 | 58.40% | 0.728 |
| RNA_virus | Togavirus | AF023424 | 58.60% | 0.702 |
| RNA_virus | Togavirus | NC_001918 | 58.90% | 0.758 |
| RNA_virus | Togavirus | AB018667 | 59.00% | 0.701 |
| RNA_virus | Togavirus | HGU44402 | 59.10% | 0.685 |
| RNA_virus | Togavirus | D90601 | 59.60% | 0.692 |
| RNA_virus | Togavirus | HGU36380 | 59.70% | 0.705 |
| RNA_virus | Togavirus | AF023425 | 60.20% | 0.794 |
| RNA_virus | Togavirus | NC_001545 | 69.60% | 1.057 |
